# Ectodermal Dysplasia Presenting as Heat Exhaustion in an Adolescent Boy

**DOI:** 10.7759/cureus.13450

**Published:** 2021-02-20

**Authors:** Giridhar Guntreddi, Jayasree Vasudevan Nair, Swayam P Nirujogi

**Affiliations:** 1 Pediatrics, SANFORD Medical Center, Bemidji, USA; 2 Pediatrics, Sutter Health, Jackson, USA; 3 Family Medicine, Tower Health Medical Group, Reading, USA

**Keywords:** ectodermal dysplasia, dehydration, hypodontia, enamel, prosthetic teeth

## Abstract

Ectodermal dysplasia (ED) is a rare heterogenous group of ectodermal disorder, which primarily affects skin, hair, nails, eccrine glands, and teeth. Hypohidrotic ED is characterized by hypotrichosis (sparseness of scalp and body hair), hypohidrosis (reduced ability to sweat), and hypodontia (congenital decrease in the number of teeth /anodontia - complete absence of teeth). Primary care physicians and dentists play a crucial role in the early diagnosis and subsequent follow ups. A careful and thorough examination of these patients will lead to accurate diagnosis. Timely involvement of a multidisciplinary team in their care including the child psychologist, dermatologist, otorhinolaryngologist, and speech therapist would avoid fatal complications and improve the overall quality of life.

In this article, we report that ED is a chronic underdiagnosed condition and can have devastating long-term complications. This is significant because with early diagnosis and prompt education of parents, patients can have better outcome in the prevention and timely management of complications such as heat exhaustion, electrolyte imbalance, heat stroke, and severe dehydration. Our case report would help clinicians familiarize with this rare condition to improve clinical acumen and better the patient outcome.

## Introduction

Ectodermal dysplasia (ED) is a rare heterogenous group of ectodermal disorder, which primarily affects skin, hair, nails, eccrine glands, and teeth. The incidence of ED is around 1:10,000 to 1:100,000 worldwide [1]. Even though more than 170 types are described in the literature, the most common syndromes in this group are hypohidrotic/anhidrotic ED, also known as Christ-Siemens-Touraine syndrome, and hidrotic ED, also known as Clouston syndrome. Hypohidrotic ED is characterized by hypotrichosis (sparseness of scalp and body hair), hypohidrosis (reduced ability to sweat), hypodontia (congenital decrease in number of teeth), and anodontia (complete absence of teeth).

## Case presentation

**Figure 1:**
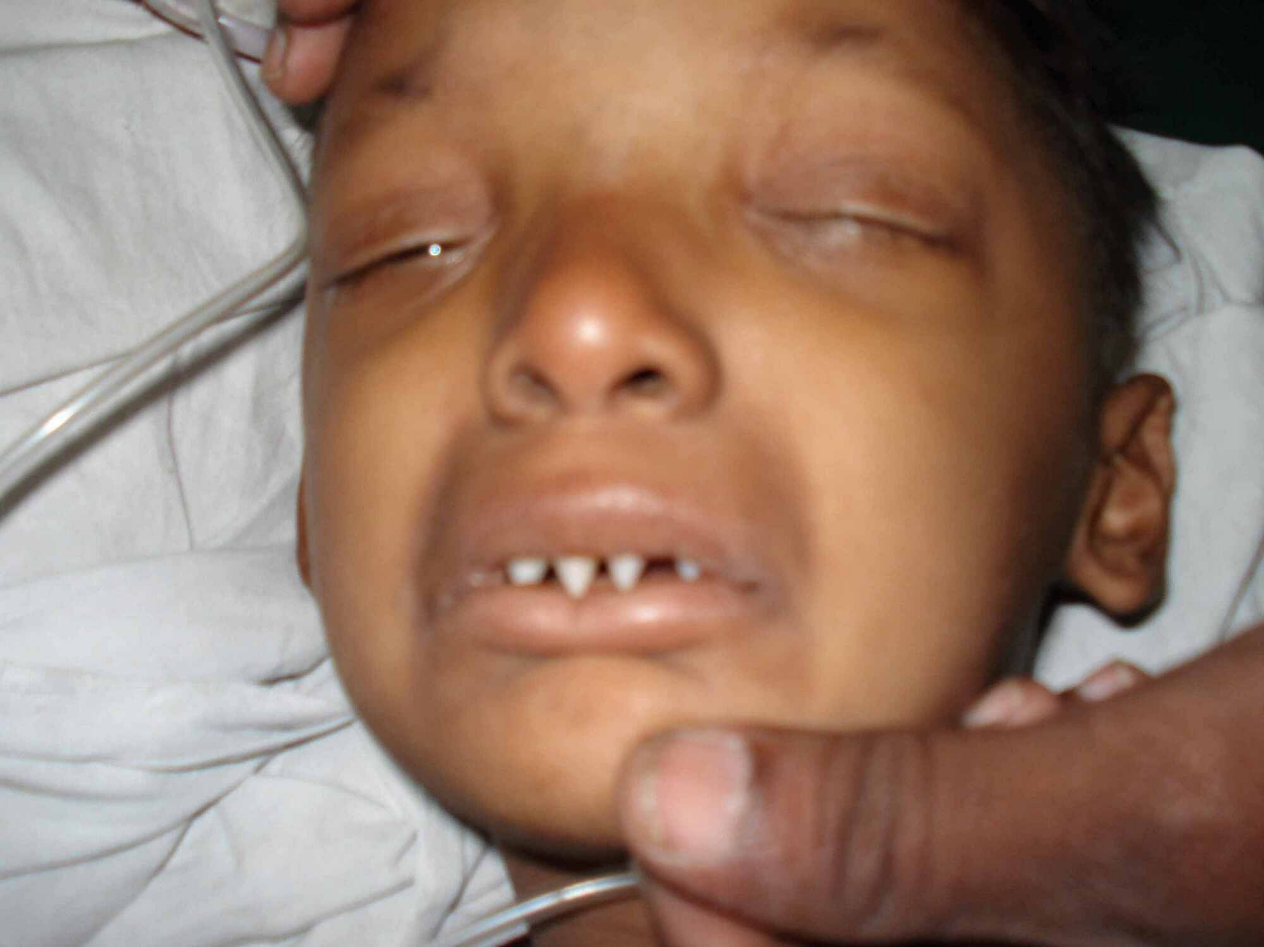
Characteristic conical teeth which are widely placed.

**Figure 2:**
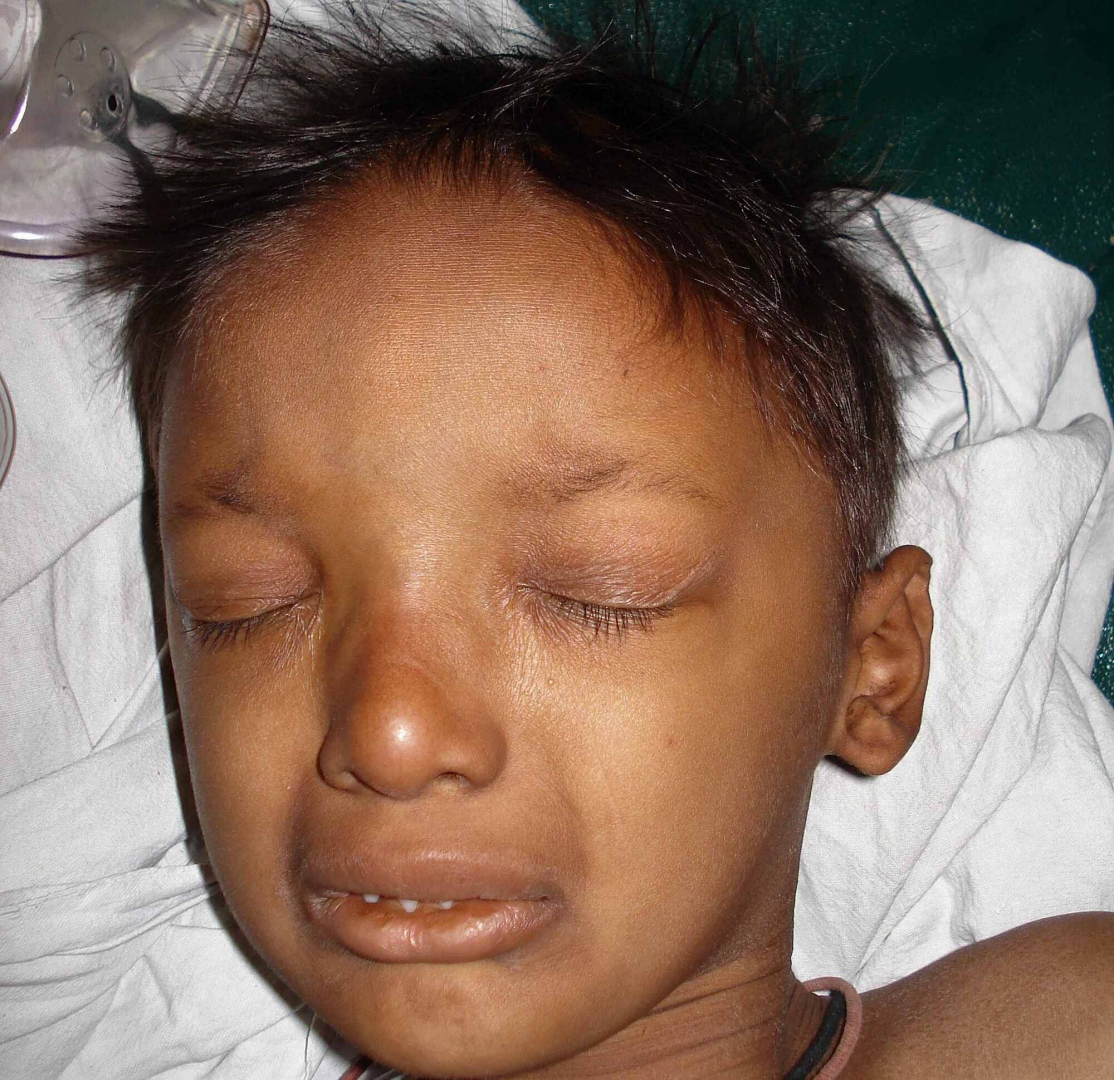
Sparse eyebrows, thin scalp hair, thick everted lips, large protruding ears, hyperpigmentation around eyes, nose, diffuse dry skin, and depressed nasal bridge.

A 11-year-old South Indian male child presented to the pediatric ER with acute onset of high-grade fever 104°F (40 degree Celsius), two episodes of nonbilious vomiting, decreased alertness, and irritability. According to his parents, he was actively playing outside in hot summer along with his friends. He collapsed while playing and had two episodes of nonbilious vomiting while coming to the hospital ER. The boy was slightly drowsy, irritable with hot red skin. His pulses were weak and thready. His vital signs are heart rate (HR) - 140 beats/min, temperature - 104°F, respiratory rate (RR) - 26 breaths/min, and blood pressure (BP) - 90/60 mmHg. On examination, he had diffuse dry skin, which is red and hot without any sweating, lips were dry and everted. His teeth were conical (Figure 1), which were widely spaced with poor enamel. His hair is thin, brittle, and sparse. He had diffuse hyperpigmentation around the eyes and nose (Figure 2). He was diagnosed with exertional heat illness secondary to his poor ability to sweat, high environmental temperature, and limited PO intake of water while playing. He was treated with cold sponging, tepid ice cube bathing, IV normal saline boluses, and maintenance IV fluids with added electrolytes. His past history is remarkable for recurrent episodes of similar fevers, dry skin, and exercise intolerance, particularly in hot weather. Also, his teeth have been abnormal since infancy. His family history was unremarkable.

Recurrent episodes of intolerance to heat/hot climates, decreased/absent sweating in hot weather, characteristic features of widely spaced conical teeth, diffuse dry and hot skin, sparse scalp hair, absent body hair prompted us towards clinical diagnosis of ED. The final diagnosis is heat exhaustion secondary to ED.

## Discussion

In most cases, hypohidrotic dysplasia shows an X-linked pattern, mostly involving males. This gene mapping to X q 12- q 13.1, encoded a ligand Ectodysplasin - A1. Female patients may show partial expression of this abnormal gene [2]. Hidrotic ED usually presents as an autosomal dominant pattern by changes in the GJB 6 gene, encoding connexin -30 and located in chromosome 13. Molecular studies found that these genes are responsible for the formation of several substrates required for the activation of the TNF - alpha related signaling pathway, the WNT - signaling pathway, and the nuclear factor - KB ( Kappa Beta) pathway, involved in ectoderm-mesoderm interaction, differentiation of ectodermal appendages, and organogenesis during the initiation of embryonic development [3].

 Oral manifestations include hypodontia (decreased teeth) or complete anodontia (absence of teeth) , abnormal shape of teeth, conical shaped teeth (Figure 1), enamel hypoplasia, maxillary retrusion, high palatal arch, and delayed eruption of permanent teeth. Due to abnormal/absent teeth, they often have masticatory difficulties, nutritional deficiencies, and speech problems. Typical facies are characterized by frontal bossing, sunken cheeks, saddle nose, thick and everted lips (Figure 2), wrinkled and hyperpigmented skin around the eyes, large and low set ears. Due to limited ability to sweat, these patients have a propensity to develop hyperthermia with physical exertion during exposure to hot environment. The scalp hair, eyebrows, and eyelashes are sparse, fine, and often lightly pigmented. They often have thick nasal secretions and thick concretions in the ear canals [4]. Other signs are short stature, eye abnormalities, dry mouth, and photophobia. Nails are often brittle, thin, and show abnormal ridging. Patients with hypohidrotic ED may suffer from low self-esteem, insecurity, and depression due to their unusual physical appearances and lack of social acceptance [5].

 Most cases of ED can be diagnosed with characteristic clinical features and dental changes. Early prenatal diagnosis can be established by DNA-based linkage analysis and genetic tests for detecting mutations in EDA/EDAR/EDAEADD [6]. In the second trimester of pregnancy, sonography and fetal skin biopsy are suitable diagnostic tests [7]. Skin biopsy shows hyperkeratosis, acanthosis with widened intercellular spaces and acantholytic keratinocytes, reduced sweat glands, sebaceous glands, and hair follicles [8]. Immunohistochemical and electron microscopic studies have demonstrated poorly developed, small desmosomes along with a reduction in the number of desmosomes in the epidermis, particularly involving the lower supra basal layer [9]. Identification of a hemizygous EDA pathogenic variant in an affected male or biallelic EDAR, EDARADD, or WNT 10 A pathogenic variants in an affected male or female confirms the diagnosis. The diagnosis of mild HED is established in a male by the identification of a heterozygous EDAR, EDARADD, or WNT10A pathogenic variant.

 There is no specific treatment for ED. These patients often need multidisciplinary care from specialties such as dentistry, dermatology, orofaciomaxillary surgery, ENT, ophthalmology, and primary care physicians for early diagnosis and subsequent follow-ups. Dental follow-ups should start around one year of age. Dental implants in the anterior portion of the mandibular arch have proven successful only in children aged seven years and older. Children with hypohidrotic ectodermal dysplasia (HED) typically need to have their dental prosthesis replaced every 2.5 years [10]. Hyposalivation will be treated by frequent sips of water, lozenges, and sour candies. Early replacement of missing teeth has a positive effect on growth and self-esteem and aids in restoring masticatory function, esthetics, and speech, ultimately leading to a better quality of life [10]. During hot weather affected individuals must have access to an adequate supply of water, cool environment, which may mean “cooling vests” air condition, a wet T shirt, and/or a spray bottle of water [11]. Other measures to prevent exertional heat illness are cold sponging, placing the child in a tub containing cold water or ice cubes, and seeking air-conditioned surroundings. The parents should be thoroughly advised to avoid indulging these children in heavy physical activities and exposure to high temperatures. ENT support may be needed for removal of ear and nasal concretions and management of epistaxis. Humidification of ambient air/cool mist vaporizers helps prevent dried secretions and concretions. Skin moisturizers would be useful for associated dry skin and eczema. Artificial tear drops will be helpful for recurrent dry eye symptoms. IV injection of recombinant EDA- A 1 to newborn dogs with X-linked HED has been found to restore the growth of the teeth, skin structures, and mucous glands. Furthermore, intra-amniotic injections of recombinant EDA- A 1 to pregnant mice partially improved the phenotype of the X-linked HED newborn mice [12].

## Conclusions

Our patient was closely monitored and treated with supportive care, cold sponging, and vigorous IV hydration. In two days, his condition was much improved. Parents were well informed and educated about the final diagnosis, possible predisposing risk factors, chronic management of eczema, dry skin, and the need for close follow-up with the dentist and primary care physician. ED is a rare but serious chronic skin disorder with lifelong complications. Primary care physicians and dentists play a crucial role in the early diagnosis and subsequent follow-ups. An understanding of fatal complications such as exertional heat illnesses and severe dehydration is vital when caring these children. A careful and thorough examination of these patients will lead to an accurate diagnosis. Early diagnosis and timely involvement of the multidisciplinary team in their care with child psychologist, dermatologist, otorhinolaryngologist, and speech therapist would avoid fatal complications and improve overall quality of life. Our case report would help clinicians familiarize with this rare condition to improve their clinical acumen and patient outcome.
